# Patients with Obesity: Baseline Body Composition, Follow-Up Retention, and Longitudinal Changes—A Single-Center Real-World Study

**DOI:** 10.3390/life16040617

**Published:** 2026-04-07

**Authors:** Banu Açmaz, Sami Bahçebaşı, Nazmiye Serap Biçer, İfakat İrem Biçer, Erdem Aydın, Mehmet Yasin Türkmen, Gökhan Açmaz

**Affiliations:** 1 Department of Internal Medicine, Kayseri City Hospital, Kayseri 38010, Turkey; banuacmaz1313@gmail.com (B.A.); doktorsami@yahoo.com (S.B.); serapbicr@gmail.com (N.S.B.); iremkrtkn@gmail.com (İ.İ.B.); erdemaydin129@gmail.com (E.A.); mehmetturkmen96@gmail.com (M.Y.T.); 2 Department of Obstetrics and Gynecology, Erciyes University, Kayseri 38039, Turkey

**Keywords:** obesity, body composition, bioelectrical impedance analysis, weight loss, follow-up studies, diabetes mellitus, Type 2

## Abstract

Aim: Using real-world data from an obesity clinic, we aimed primarily to define the body composition phenotype associated with increasing obesity severity and to quantify follow-up retention and clinically meaningful weight loss. As a secondary exploratory objective, we also examined how diabetes mellitus (DM) relates to these patterns beyond BMI. Methods: Among 5350 screened adults in the Obesity Clinic database, 2879 eligible individuals were included in the baseline cross-sectional analyses, including a non-obese comparison subgroup with BMI < 30 kg/m^2^. The longitudinal cohort comprised 730 patients with obesity who returned for regular follow-up and had repeat BIA measurements at least 6 months after baseline. Patients were classified according to BMI and glycemic status. Results: Increasing BMI was associated with higher glucose, HbA1c, uric acid, triglyceride, C-reactive protein, and leukocyte levels and with lower HDL cholesterol. Body composition deterioration was driven predominantly by disproportionate increases in visceral fat score, fat mass, and total fat-to-muscle ratio rather than by parallel gains in muscle compartments. As obesity severity increased, the proportion of patients with diabetes also increased. Only 29.3% of the baseline obesity cohort achieved regular 6-month follow-up, and 79.0% of those followed lost less than 5% of baseline body weight. In age- and sex-adjusted analyses, the most pronounced DM-related body composition differences were observed in the BMI 30–39.9 kg/m^2^ group, particularly through higher waist-to-height ratio and total fat-to-muscle ratio. Conclusions: This study shows that increasing obesity severity is accompanied by a more adverse metabolic profile and a body composition pattern characterized by greater adiposity. Within this framework, diabetes-related body composition differences were not homogeneous across BMI categories and appeared to be most evident in the BMI 30–39.9 kg/m^2^ group. The weaker association observed in the BMI ≥ 40 kg/m^2^ group may be related to the reduced discriminative value of waist-based measures in severe obesity, where fat accumulation may extend across multiple compartments. Given the low continuity of follow-up and the limited rates of clinically meaningful weight loss, these findings support a more refined phenotyping approach in obesity management that incorporates body composition and fat distribution in addition to BMI.

## 1. Introduction

The global burden of obesity continues to rise at an unprecedented pace, representing one of the most pressing public health challenges of the 21st century. According to the Global Burden of Disease (GBD) 2021 study, the prevalence of overweight and obesity has increased substantially since 1990 and is projected to affect the majority of the adult population worldwide by 2050 [1]. Beyond its growing prevalence, obesity is characterized by considerable metabolic heterogeneity, with marked interindividual variation in body composition and associated cardiometabolic risk that cannot be fully captured by body mass index (BMI) alone. Therefore, a more comprehensive assessment of adiposity distribution, particularly central fat accumulation in relation to muscle compartments, is essential for more accurate risk stratification.

Obesity is increasingly recognized as a complex, multifactorial, and progressive disease arising from the interplay of genetic susceptibility, environmental exposures, and behavioral factors, rather than from a simple imbalance between energy intake and expenditure. Importantly, accumulating evidence indicates that individuals with similar BMI levels may exhibit markedly different metabolic profiles and clinical outcomes, underscoring the limited ability of BMI to reflect underlying biological risk. This heterogeneity is largely driven by differences in fat distribution, ectopic fat deposition, and adipose tissue function, all of which are more closely associated with cardiometabolic complications than total body weight alone. Consequently, contemporary perspectives emphasize the need to move beyond weight-centered classifications toward a more integrated assessment of adiposity and its clinical consequences [2,3].

In this context, anthropometric indicators reflecting central adiposity, particularly waist circumference and waist-to-height ratio, have emerged as practical tools that provide clinically meaningful information beyond BMI for the assessment of cardiometabolic risk associated with abdominal fat accumulation. In fact, the NICE guideline recommends the use of waist-to-height ratio alongside BMI in adults with BMI < 35 kg/m^2^ and classifies values of 0.5–0.59 as indicating increased health risk and values ≥ 0.6 as indicating central adiposity associated with a substantially higher health risk [4]. 

For a more detailed evaluation of body composition, bioelectrical impedance analysis (BIA) represents a practical and non-invasive method that extends obesity phenotyping beyond body weight alone by providing additional information on fat mass, body fat percentage, fat-free mass, muscle compartments, and body fluid distribution. Moreover, BIA-derived parameters have been shown to be clinically useful in the assessment and follow-up of body composition in individuals with obesity; however, because these measurements may be influenced by device-specific and methodological characteristics, their interpretation requires appropriate standardization of measurement conditions [5].

Obesity management is based on a chronic disease model that requires not only initial weight reduction but also long-term care, sustained follow-up, and comprehensive management of accompanying cardiometabolic risk [6]. One of the major challenges in clinical practice is maintaining patient retention during long-term follow-up and determining whether observed changes extend beyond body weight to reflect meaningful alterations in body composition. Nevertheless, real-world studies that simultaneously evaluate baseline body composition, follow-up adherence, and treatment-related longitudinal changes remain relatively limited. This issue is particularly relevant in individuals with coexisting type 2 diabetes, in whom treatment-related changes in HbA1c may occur alongside changes in waist circumference and body composition, suggesting a close interplay among glycemic status, central adiposity, and compositional response [7].

In the present study, we aimed to use real-world data from patients followed within the routine clinical care process of an obesity center to characterize baseline body composition features, evaluate follow-up retention and weight-loss success, and describe longitudinal changes in anthropometric and body composition parameters. In doing so, the study was designed to move beyond a static BMI-based classification and to provide a more comprehensive clinical framework that jointly evaluates central adiposity, BIA-based parameters, and weight-loss success using data that reflect the real-world practice of an obesity center.

## 2. Materials and Methods

### 2.1. Study Design and Setting

This study was designed as a single-center, retrospective, real-world cohort study based on data from patients with obesity who were followed as part of routine clinical care at the Obesity Clinic of Kayseri City Hospital. The clinic operates within a multidisciplinary obesity management framework, with patient follow-up conducted by an outpatient unit including one dietitian, one psychologist, and two internal medicine specialists. Body composition assessments were performed in a dedicated measurement room within the clinic using a Tanita MC-780MA segmental body composition analyzer (Tanita Corp., Tokyo, Japan). This device is based on multi-frequency bioelectrical impedance analysis (BIA) technology and enables segmental body composition assessment for clinical evaluation and weight-loss monitoring. The primary aim of the study was to characterize baseline body composition patterns across obesity severity and to evaluate follow-up adherence, weight-loss success, and longitudinal changes in body composition in a real-world obesity clinic setting.

### 2.2. Study Population

A total of 5350 adults recorded in the Obesity Clinic database of Kayseri City Hospital between 2023 and 2025 were screened. Of these, 2879 individuals with accessible electronic medical records, available clinical, biochemical, anthropometric, and body composition data for baseline assessment, and who met the prespecified inclusion and exclusion criteria were included in the baseline analyses. Within this baseline analytic cohort, participants with a BMI < 30 kg/m^2^ and available BIA-based body composition measurements constituted the non-obese comparison group; the same general exclusion criteria applied to the obesity group were also used for this subgroup.

**Figure 1:**
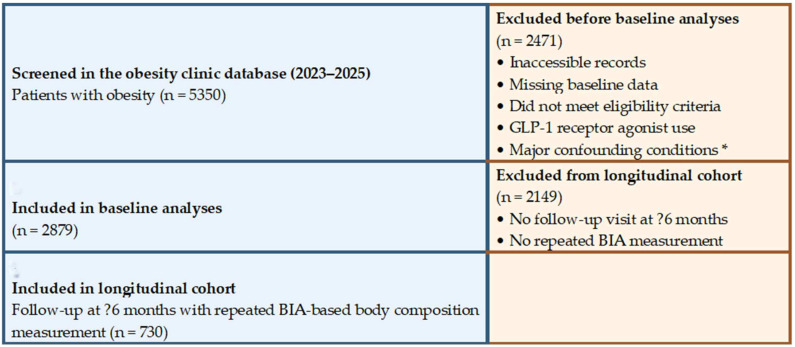
Flow diagram of patient selection and cohort formation. A total of 5350 patients with obesity recorded in the obesity clinic database between 2023 and 2025 were screened. After application of the inclusion and exclusion criteria, 2879 patients were included in the baseline analyses and 730 in the longitudinal cohort. * Major confounding conditions included malignancy, active infection or inflammatory disease, advanced organ failure, and uncontrolled thyroid dysfunction.

For the longitudinal analyses, only patients with obesity from the baseline obesity cohort who attended a follow-up visit at least 6 months after the baseline assessment as part of routine obesity follow-up and had a repeated BIA-based body composition measurement at that visit were included; the non-obese comparison group was not included in these analyses. A total of 730 patients met these criteria and constituted the longitudinal obesity cohort. The patient selection process and cohort formation are summarized in Figure 1.

### 2.3. Follow-Up Protocol and Medical Nutrition Therapy

Routine follow-up in the obesity clinic was based on approximately monthly evaluations conducted in the obesity nutrition outpatient clinic. The follow-up process was managed by the dietitian and two internal medicine specialists, with psychological support provided when clinically indicated. Medical nutrition therapy was delivered as an individualized energy-restricted dietary intervention, planned in accordance with current guideline recommendations and tailored to patients’ eating habits, daily routines, clinical characteristics, and estimated energy requirements [8]. At each follow-up visit, body weight was reassessed, adherence to the dietary plan was reviewed, and the nutritional intervention was modified as needed according to clinical requirements. For the purposes of the present study, the follow-up visit used for longitudinal analyses was defined as an eligible control visit performed at least 6 months after the baseline assessment and including a repeated BIA-based body composition measurement. Accordingly, the longitudinal cohort consisted of patients who had at least one such eligible follow-up visit in addition to the baseline evaluation, allowing serial assessment of body composition.

### 2.4. Inclusion and Exclusion Criteria

Patients with obesity who had available baseline demographic, laboratory, anthropometric, and BIA-based body composition data were included in the baseline cohort. For inclusion in the longitudinal analyses, patients were additionally required to have attended a follow-up visit at least 6 months after the baseline assessment and to have undergone repeated BIA measurement at that visit.

In the overall study cohort, patients receiving glucagon-like peptide-1 receptor agonists (GLP-1 RAs) were excluded because these therapies may exert substantial effects on body weight and body composition that cannot be interpreted independently of treatment exposure. This approach was adopted to allow a more homogeneous evaluation of baseline characteristics and longitudinal changes, given the known effects of GLP-1-based therapies on fat mass, abdominal/visceral fat distribution, and total body weight [9,10].

In addition, patients with malignancy, active infection or inflammatory disease, advanced organ failure, or uncontrolled thyroid dysfunction, all of which could independently affect body weight and body composition, were excluded.

### 2.5. Data Collection and Clinical Variables

Demographic and clinical data were obtained from electronic medical records. Recorded variables included age, sex, glycemic status, and follow-up information. Baseline laboratory assessment included glucose, HbA1c, uric acid, aspartate aminotransferase (AST), alanine aminotransferase (ALT), estimated glomerular filtration rate (eGFR), total cholesterol, LDL cholesterol, HDL cholesterol, triglycerides, non-HDL cholesterol, C-reactive protein (CRP), thyroid-stimulating hormone (TSH), leukocyte count, hemoglobin, and platelet count.

### 2.6. Anthropometric and Body Composition Measurements

Anthropometric assessment included body weight, height, and waist circumference. Body mass index (BMI) was calculated as body weight in kilograms divided by height in meters squared (kg/m^2^). Waist-to-height ratio (WHtR) was calculated by dividing waist circumference by height. Body composition was assessed using a BIA system, and the following BIA-derived parameters were analyzed at baseline and follow-up visits: body weight, BMI, fat mass (kg), body fat percentage, muscle mass (kg), skeletal muscle mass (kg), visceral fat score, and extracellular water-to-total body water ratio (ECW/TBW). The total fat-to-muscle ratio was not a device-generated parameter; rather, it was calculated by the investigators using BIA-derived fat mass and muscle mass values according to the following formula: fat-to-muscle ratio = fat mass (kg)/muscle mass (kg), and was analyzed as a raw ratio.

### 2.7. Definition of Study Groups and Weight-Loss Categories

Patients were categorized into three prespecified analytic BMI groups: BMI <30 kg/m^2^, BMI 30–39.9 kg/m^2^, and BMI ≥ 40 kg/m^2^ [11]. This grouping was not intended to replace the standard BMI classification, but rather to provide a pragmatic analytical framework that reflects increasing obesity severity while ensuring adequate group sizes for statistical comparisons. Patients were also classified as normoglycemic, prediabetic, or diabetic on the basis of baseline glycemic assessment and available clinical records, according to the diagnostic and classification approach outlined in the ADA 2025 Standards of Care [12]. Weight-loss success during follow-up was evaluated according to the percentage change in body weight at the follow-up visit relative to baseline body weight. Based on clinically meaningful weight-loss thresholds, patients were further categorized into three groups: <5% weight loss, 5% to <10% weight loss, and ≥10% weight loss [13].

### 2.8. Outcomes

The primary outcomes of the study were as follows: (i) characterization of clinical, metabolic, and body composition features across baseline BMI categories; (ii) assessment of follow-up adherence among patients eligible for longitudinal analysis; (iii) classification of weight-loss success according to the percentage change in body weight; and (iv) evaluation of changes in anthropometric and BIA-based body composition parameters in the longitudinal cohort.

### 2.9. Statistical Analysis

Statistical analyses were performed using IBM SPSS Statistics for Windows, version 22.0 (IBM Corp., Armonk, NY, USA). Continuous variables were presented as median (interquartile range [IQR]) according to their distributional characteristics, whereas categorical variables were expressed as number and percentage.

Comparisons of continuous variables across BMI categories were performed using the Kruskal–Wallis test. When the overall comparison was significant, pairwise group comparisons were conducted using the Dunn–Bonferroni post hoc test. Categorical variables were compared using the chi-square test; when appropriate, pairwise comparisons of row proportions were evaluated using the Bonferroni-adjusted z test.

To examine the associations between BMI and selected metabolic parameters, multivariable linear regression models adjusted for age and sex were constructed. Similarly, within BMI categories, the independent associations between the presence of diabetes mellitus and selected body composition parameters were evaluated using linear regression models adjusted for age and sex. Regression results were reported as unstandardized regression coefficients (B), 95% confidence intervals, and *p* values. Relative percentage changes in selected body composition parameters were calculated using the BMI < 30 kg/m^2^ group as the reference according to the following formula: [(value in the higher BMI group—value in the BMI < 30 kg/m^2^ group)/value in the BMI < 30 kg/m^2^ group] × 100. In all analyses, a two-sided *p* value of <0.05 was considered statistically significant.

### 2.10. Ethical Approval

Ethical approval for the study was obtained from the Non-Interventional Clinical Research Ethics Committee of Kayseri City Hospital (Decision No. 782; Date: 3 February 2026). The study was retrospective and observational in design, and all data were analyzed in anonymized form. The study was conducted in accordance with the principles of the Declaration of Helsinki.

## 3. Results

A total of 2879 patients with obesity who met the prespecified eligibility criteria were included in the baseline analyses. Baseline clinical and metabolic characteristics across the prespecified analytic BMI categories are presented in Table 1. Age differed significantly across BMI groups, increasing progressively from the BMI < 30 kg/m^2^ group to the BMI 30–39.9 kg/m^2^ and BMI ≥ 40 kg/m^2^ groups, whereas sex distribution was similar across categories. Higher BMI categories were associated with progressively higher glucose, HbA1c, uric acid, triglyceride, C-reactive protein, and leukocyte levels, as well as lower HDL cholesterol levels; these associations remained significant after adjustment for age and sex. ALT and total cholesterol also differed significantly across BMI groups after adjustment, whereas AST, hemoglobin, LDL cholesterol, eGFR, TSH, and non-HDL cholesterol were not independently associated with BMI category.

**Figure 2:**
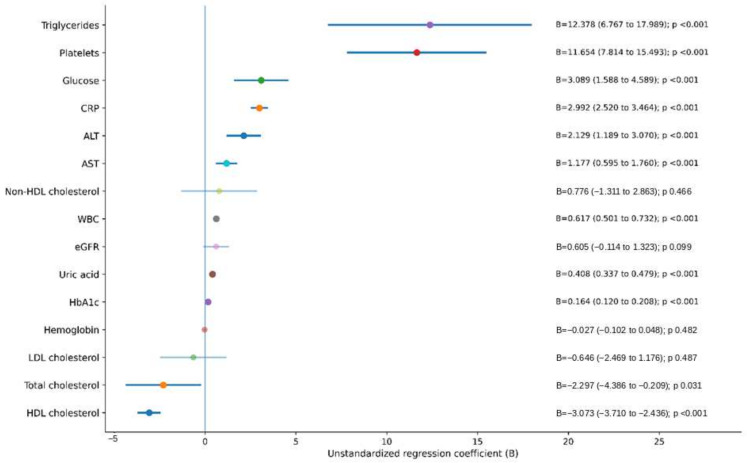
Forest plot of age- and sex-adjusted associations between BMI category and selected metabolic parameters. Dots represent unstandardized regression coefficients (B), and horizontal lines represent 95% confidence intervals. Estimates were derived from multivariable linear regression models adjusted for age and sex. Positive coefficients indicate higher values with increasing BMI category, whereas negative coefficients indicate lower values.

In age- and sex-adjusted linear regression analyses, BMI was independently and positively associated with triglycerides, platelet count, glucose, CRP, ALT, AST, leukocyte count, uric acid, and HbA1c. The strongest positive associations were observed for triglycerides (B = 12.38) and platelet count (B = 11.65), followed by glucose (B = 3.09), CRP (B = 2.99), ALT (B = 2.13), AST (B = 1.18), leukocyte count (B = 0.62), uric acid (B = 0.41), and HbA1c (B = 0.16). In contrast, total cholesterol and HDL cholesterol were inversely associated with BMI, with coefficients of −2.30 and −3.07, respectively. No statistically significant associations were observed between BMI and non-HDL cholesterol, eGFR, hemoglobin, or LDL cholesterol (Figure 2).

**Figure 3:**
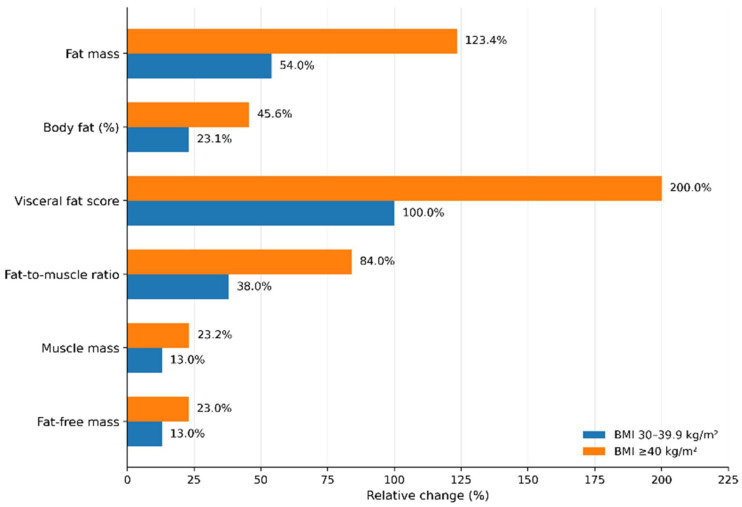
Relative changes in selected body composition parameters across BMI categories. Relative percentage changes were calculated using the BMI <30 kg/m^2^ group as the reference category. Adiposity-related parameters showed larger relative increases than muscle-related parameters across higher BMI categories.

All body composition parameters differed significantly across BMI categories (Figure 3). Using the BMI < 30 kg/m^2^ group as the reference, the largest relative increases were observed in visceral fat score, fat mass, and fat-to-muscle ratio. In the BMI 30–39.9 kg/m^2^ and BMI ≥ 40 kg/m^2^ groups, visceral fat score increased by 100.0% and 200.0%, fat mass by 54.0% and 123.4%, and fat-to-muscle ratio by 38.0% and 84.0%, respectively. In contrast, increases in muscle mass and fat-free mass were more modest, reaching 13.0% and 23.2% for muscle mass and 13.0% and 23.0% for fat-free mass, respectively. Overall, higher BMI categories were characterized by proportionally greater expansion of fat compartments than of muscle compartments.

The distribution of glycemic categories across BMI groups is presented in Table 2.

The distribution of glycemic categories differed significantly across BMI groups (χ^2^ = 168.2, df = 4, *p* < 0.001) (Table 2). In the BMI < 30 kg/m^2^ group, normoglycemia was the predominant category (76.1%), followed by prediabetes (19.0%) and diabetes (4.9%), with all pairwise comparisons being significant. In the BMI 30–39.9 kg/m^2^ group, normoglycemia remained the most frequent category (56.6%), followed by prediabetes (35.6%) and diabetes (7.8%), and again all pairwise comparisons were significant. In the BMI ≥ 40 kg/m^2^ group, the proportions of normoglycemia and prediabetes were 41.7% and 40.4%, respectively, and did not differ significantly from each other, whereas diabetes was significantly more frequent (17.9%) than both. Overall, increasing BMI was associated with a progressive decrease in the proportion of normoglycemic patients and an increase in the proportions of prediabetes and diabetes.

**Figure 4:**
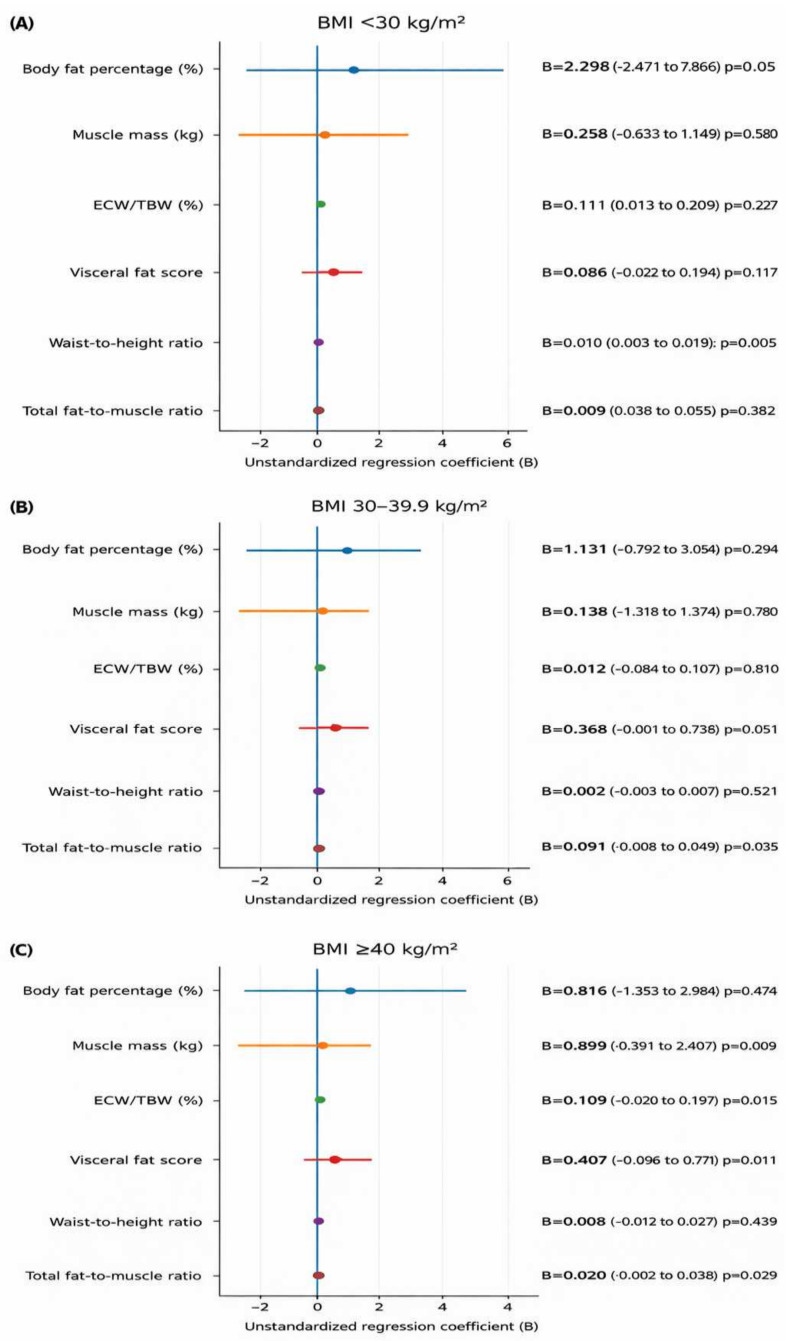
Age- and sex-adjusted associations of diabetes status with body composition parameters within BMI categories. (**A**) BMI < 30 kg/m^2^; (**B**) BMI 30–39.9 kg/m^2^; (**C**) BMI ≥ 40 kg/m^2^. Forest plots show the unstandardized regression coefficients (B) and 95% confidence intervals for the association between diabetes status and selected body composition parameters in participants with diabetes, whereas negative coefficients indicate lower values. The vertical reference line represents the null value (B = 0). Estimates were derived from linear regression models adjusted for age and sex.

After examining the distribution of glycemic categories across BMI groups, the association between diabetes status and body composition was further evaluated within BMI categories using age- and sex-adjusted regression models (Figure 4). No statistically significant independent associations were observed between diabetes status and the selected body composition parameters in the BMI < 30 kg/m^2^ and BMI ≥ 40 kg/m^2^ groups. In contrast, within the BMI 30–39.9 kg/m^2^ group, diabetes status was independently associated with a higher waist-to-height ratio (B = 0.011, 95% CI 0.001 to 0.021; *p* = 0.032) and a higher total fat-to-muscle ratio (B = 0.021, 95% CI 0.001 to 0.040; *p* = 0.035). In the same group, a positive trend was also observed for visceral fat score, although this association did not reach statistical significance (B = 0.384, 95% CI -0.001 to 0.769; *p* = 0.051).

For the longitudinal analyses, only patients with obesity who were under follow-up in the Obesity Clinic and had a repeated BIA-based body composition measurement at a follow-up visit performed at least 6 months after baseline were included; the non-obese comparison group was not included in these analyses. A total of 730 patients met these criteria, corresponding to 29.3% of the baseline obesity cohort with BMI ≥ 30 kg/m^2^. Within this longitudinal obesity cohort, 634 patients (86.8%) were female and 96 (13.2%) were male. When categorized according to percentage weight loss during follow-up, 577 patients (79.0%) achieved < 5% weight loss relative to baseline body weight, 126 (17.3%) achieved 5% to <10% weight loss, and 27 (3.7%) achieved ≥ 10% weight loss.

## 4. Discussion

In this large real-world cohort of patients with obesity, worsening BMI category was accompanied by progressive deterioration in glycemic parameters, the atherogenic lipid profile, and inflammatory markers, together with a shift in body composition toward greater adiposity. The increases in waist-to-height ratio, visceral fat score, and total fat-to-muscle ratio suggest that increasing obesity severity reflects not merely a quantitative gain in body weight but also a more adverse metabolic and compositional phenotype. These findings are consistent with previous evidence indicating that obesity-related cardiometabolic risk is determined not only by total body weight, but also by fat distribution and the relative balance between fat and muscle compartments. Notably, Raheem et al. showed that MRI-measured visceral adiposity was associated, independently of BMI or subcutaneous fat, with triglyceride-rich lipoproteins, fatty acids, inflammation, glucose, and intermediary metabolites, and that this pattern resembled metabolomic profiles predictive of type 2 diabetes and myocardial infarction [14]. Similarly, Coral et al. reported that discordant biomarker profiles characterized by higher TG, lower HDL, higher LDL, higher CRP, or higher fasting glucose were associated with differences in coronary disease prevalence and future major adverse cardiovascular event risk even at the same BMI level [15]. Within this framework, the progressive metabolic deterioration observed in our cohort with increasing BMI and adiposity supports the concept that visceral fat accumulation contributes to cardiometabolic risk through insulin resistance and proinflammatory activity.

Another notable finding of our study was that, after adjustment for age and sex, the association between diabetes and body composition varied across BMI categories and was particularly evident in the BMI 30–39.9 kg/m^2^ group. This observation is consistent with imaging-based data suggesting that distinct fat distribution patterns may confer different metabolic risk profiles even within the same BMI range. In this regard, Linge et al. reported that, among individuals with BMI 30–40 kg/m^2^, visceral adiposity and liver fat were positively associated with type 2 diabetes, whereas abdominal subcutaneous fat showed an inverse association, and these relationships remained significant after adjustment for age, sex, and BMI [16]. Ross et al. further emphasized that BMI alone does not adequately capture cardiometabolic risk, that waist circumference provides additional and independent information, and that once the expandability of subcutaneous adipose tissue is exceeded, excess fat may be diverted to visceral and ectopic depots, thereby promoting a more adverse metabolic profile [17]. Likewise, Agrawal et al. demonstrated that, even at the same BMI level, visceral, abdominal subcutaneous, and gluteofemoral fat depots may show divergent or even opposite associations with cardiometabolic diseases [18]. In light of these data, one possible explanation for our findings is that central adiposity may play a more prominent role during the earlier and intermediate stages of weight gain, whereas at very high BMI levels, further fat accumulation may be redistributed across multiple compartments, thereby reducing the discriminative value of waist-based indices. These findings also suggest that obesity should not be considered a metabolically uniform condition across all BMI levels. Accordingly, the more apparent association between diabetes and abdominal adiposity in the BMI 30–39.9 kg/m^2^ group, and the weaker association in the BMI ≥ 40 kg/m^2^ group, may reflect phenotypic saturation or a ceiling effect in severe obesity. In this context, “phenotypic saturation” refers to a state in which adverse adiposity-related features are already highly expressed across multiple compartments, thereby limiting the ability of individual anthropometric markers to further distinguish phenotypic variability.

Another important longitudinal finding was that, despite the substantial cardiometabolic risk burden of this population, follow-up continuity and clinically meaningful weight loss remained limited. Only a subset of the baseline cohort had a repeat BIA-based body composition assessment after at least 6 months, and most followed patients achieved <5% weight loss, suggesting that long-term retention and sustained engagement remain major challenges in real-world obesity care. This observation is consistent with the findings of Chen et al., who showed that a higher number of clinic visits and a longer duration of attendance in a multidisciplinary obesity clinic significantly increased the likelihood of achieving ≥5% weight loss, whereas only a limited proportion of patients maintained such weight loss over the long term [19]. Similarly, Perna et al. reported dropout rates of 44.4% at 6 months and 68.5% at 12 months after a multidisciplinary obesity program [20]. Thus, the low follow-up rate observed in our cohort should not be interpreted solely as attrition, but also as an important real-world feature of chronic obesity care. Taken together, these data suggest that the presence of diabetes and other comorbidities alone does not ensure adequate follow-up adherence; rather, without structured, accessible, and sustainable care pathways, treatment continuity remains fragile even in high-risk patients.

Taken together, these findings suggest that obesity care in routine practice is limited not only by adverse adiposity patterns and metabolic risk, but also by poor long-term retention. Overall, our results support the need for an approach that goes beyond BMI alone and incorporates body composition into the clinical evaluation of obesity. The marked female predominance in our cohort may reflect sex-related differences in treatment-seeking behavior and body image dissatisfaction rather than prevalence alone, and these factors should be specifically examined in future real-world studies.

## 5. Limitations

This study has several limitations. First, its single-center and observational design precludes causal inference. Second, the longitudinal analyses could only be performed in a subset of the baseline cohort, and the limited follow-up continuity together with the low proportion of patients achieving clinically meaningful weight loss may have reduced generalizability. Although the present study was not designed to identify independent predictors of dropout or limited weight-loss response, these questions are clinically important and should be addressed in future real-world studies using multivariable modeling approaches. In addition, body composition was assessed using bioelectrical impedance analysis (BIA) with a single device, which may be influenced by hydration status and other method-specific technical limitations. Finally, the proposed explanation for the weaker association observed in severe obesity, including possible phenotypic saturation or reduced discriminatory value of waist-based measures, was not directly tested with imaging-based methods and should therefore be considered hypothesis-generating.

## 6. Conclusions

In conclusion, increasing obesity severity was accompanied by worsening metabolic risk and a body composition pattern characterized by greater adiposity. The relationship between diabetes and body composition was not homogeneous across BMI categories and was most apparent in the BMI 30–39.9 kg/m^2^ group. The low continuity of follow-up and the limited achievement of clinically meaningful weight loss further highlight the gap between cardiometabolic risk burden and effective long-term obesity care in real-world practice. These findings support a more refined clinical approach to obesity and diabetes that incorporates body composition and fat distribution in addition to BMI.

## Figures and Tables

**Table 1:** Clinical and metabolic characteristics according to BMI categories.

Variable	BMI < 30 kg/m^2^ (n = 385)	30–39.9 kg/m^2^ (n = 1573)	≥40 kg/m^2^ (n = 921)	*p*	*p*-Adj
Age (years)	34 (26–43) ^a^	37 (29–46) ^b^	42 (31–52) ^c^	<0.001	—
Male, n (%)	81 (21.0)	311 (19.8)	162 (17.6)	0.259	—
Female, n (%)	304 (79.0)	1262 (80.2)	759 (82.4)		
Glucose (mg/dL)	88 (82–93) ^a^	90 (84–97) ^b^	93 (86–103) ^c^	<0.001	<0.001
HbA1c (%)	5.4 (5.2–5.6) ^a^	5.6 (5.3–5.8) ^b^	5.7 (5.4–6.1) ^c^	<0.001	<0.001
Uric acid (mg/dL)	4.6 (3.8–5.6) ^a^	5.1 (4.3–5.9) ^b^	5.5 (4.6–6.4) ^c^	<0.001	<0.001
AST (U/L)	18 (15–22)	18 (15–22)	18 (15–23)	0.078	—
ALT (U/L)	16 (12.5–23) ^a^	19 (14–27) ^b^	19 (14–27) ^b^	<0.001	<0.001
eGFR (mL/min/1.73 m^2^)	113 (103–122) ^c^	112 (101–120) ^b^	108 (96–118) ^a^	<0.001	0.116
Total cholesterol (mg/dL)	178.5 (159–204) ^a^	184.5 (161–209) ^b^	187 (163–211) ^b^	0.035	0.022
LDL cholesterol (mg/dL)	117 (96–136) ^a^	121 (99–142) ^b^	121 (102–143) ^b^	0.016	0.539
HDL cholesterol (mg/dL)	51 (43–59) ^c^	46 (39–54) ^b^	45 (38–53) ^a^	<0.001	<0.001
Triglycerides (mg/dL)	98 (71–135) ^a^	124 (90–173) ^b^	134 (102–183) ^c^	<0.001	<0.001
Non-HDL cholesterol (mg/dL)	130 (107–153) ^a^	138 (115–163) ^b^	140 (117–163) ^b^	<0.001	0.054
CRP (mg/L)	1.7 (0.8–3.4) ^a^	3.8 (1.9–7.1) ^b^	6.8 (3.9–11.6) ^c^	<0.001	<0.001
TSH (mIU/L)	1.86 (1.35–2.60) ^a^	1.88 (1.32–2.62) ^a^	2.09 (1.46–2.89) ^b^	<0.001	0.302
WBC (×10^3^/µL)	7.19 (6.23–8.24) ^a^	7.73 (6.63–9.01) ^b^	8.18 (6.99–9.76) ^c^	<0.001	<0.001
Hemoglobin (g/dL)	13.95 (13.1–14.9)	13.8 (12.9–14.8)	13.9 (12.9–14.9)	0.213	—
Platelets (×10^3^/µL)	276 (242–319) ^a^	289 (249–334) ^b^	293 (252–343) ^c^	<0.001	<0.001

Data are presented as median (interquartile range) for continuous variables and n (%) for categorical variables. Group comparisons for continuous variables were performed using the Kruskal–Wallis test. When the overall comparison was significant, pairwise post hoc comparisons were performed using the Dunn–Bonferroni test; different superscript letters (a, b, c) indicate statistically significant differences between BMI groups. Categorical variables were compared using the chi-square test. The *p*-adj column represents *p*-values adjusted for age and sex for continuous variables, except for age itself. Adjusted *p*-values were not calculated for categorical variables. Abbreviations: BMI, body mass index; HbA1c, glycated hemoglobin; AST, aspartate aminotransferase; ALT, alanine aminotransferase; eGFR, estimated glomerular filtration rate; LDL, low-density lipoprotein; HDL, high-density lipoprotein; CRP, C-reactive protein; TSH, thyroid-stimulating hormone; WBC, white blood cell count.

**Table 2:** Distribution of glycemic categories according to BMI groups.

Glycemic Group	BMI < 30 kg/m^2^ (n = 385)	BMI 30–39.9 kg/m^2^ (n = 1573)	BMI ≥ 40 kg/m^2^ (n = 921)	*p*
Normoglycemic	293 (76.1%) ^a^	891 (56.6%) ^a^	384 (41.7%) ^a^	
Prediabetes	73 (19.0%) ^b^	560 (35.6%) ^b^	372 (40.4%) ^a^	
Diabetes	19 (4.9%) ^c^	122 (7.8%) ^c^	165 (17.9%) ^b^	<0.001

Values are presented as n (%). Percentages represent column percentages within each BMI group. Group differences were assessed using the chi-square test. When the overall test was significant, pairwise post hoc comparisons of glycemic-category proportions within each BMI group were performed using z-tests with Bonferroni correction. Different superscript letters indicate statistically significant differences within the same column, whereas identical superscript letters indicate no significant difference. Overall chi-square test: χ^2^ = 168.2, df = 4, *p* < 0.001.

## Data Availability

The data that support the findings of this study are available from the corresponding author upon reasonable request.
